# Effect of Penetration Enhancer Containing Vesicles on the Percutaneous Delivery of Quercetin through New Born Pig Skin

**DOI:** 10.3390/pharmaceutics3030497

**Published:** 2011-08-12

**Authors:** Maura Chessa, Carla Caddeo, Donatella Valenti, Maria Manconi, Chiara Sinico, Anna Maria Fadda

**Affiliations:** Dipartimento Farmaco Chimico Tecnologico, University of Cagliari, Via Ospedale 72, Cagliari, Italy

**Keywords:** liposome, quercetin, penetration enhancer, skin permeation, rheology, confocal laser scanning microscopy, bioflavonoid

## Abstract

Quercetin (3,3′,4′,5,7-pentahydroxyflavone) exerts multiple pharmacological effects: anti-oxidant activity, induction of apoptosis, modulation of cell cycle, anti-mutagenesis, and anti-inflammatory effect. In topical formulations quercetin inhibits oxidative skin damage and the inflammatory processes induced by solar UV radiation. In this work, quercetin (2 mg/mL) was loaded in vesicular Penetration Enhancer containing Vesicles (PEVs), prepared using a mixture of lipids (Phospholipon^®^ 50, P50) and one of four selected hydrophilic penetration enhancers: Transcutol^®^ P, propylene glycol, polyethylene glycol 400, and Labrasol^®^ at the same concentration (40% of water phase). Photon Correlation Spectroscopy results showed a mean diameter of drug loaded vesicles in the range 80–220 nm. All formulations showed a negative surface charge and incorporation efficiency in the range 48–75%. Transmission Electron Microscopy confirmed that size and morphology varied as a function of the used penetration enhancer. The influence of PEVs on *ex vivo* quercetin (trans)dermal delivery was evaluated using Franz-type diffusion cells, new born pig skin and Confocal Laser Scanning Microscopy. Results showed that drug delivery is affected by the penetration enhancer used in the PEVs' formulation.

## 1. Introduction

Quercetin (3,3′,4′,5,7-pentahydroxyflavone, QUE) is a bioflavonoid, profuse in nature in plant food sources. It exerts multiple pharmacological effects, including potent anti-oxidant activity *in vivo*, induction of apoptosis, modulation of cell cycle, anti-mutagenesis, inhibition of angiogenesis, and anti-inflammatory effects. Recently, it has been reported that the topical application of quercetin inhibits oxidative skin damage and the inflammatory processes induced by solar UV radiation [1]. Topical use of quercetin is frequently hampered by its low skin permeability and poor solubility in aqueous media, which make the development of pharmaceutical formulations difficult. Different strategies, such as prodrug [2] and microemulsion [3], have been used to improve quercetin topical delivery.

In this work, novel quercetin vesicular formulations are presented for skin delivery. With the aim of incorporating quercetin into phospholipid vesicles, four different hydrophilic penetration enhancers (PEs), essential to improve quercetin solubility in water due to their high solubilizing power, were selected and used to formulate quercetin-loaded Penetration Enhancer containing Vesicles (PEVs). PEV formulations were prepared using 60 mg/mL of a mixture of soy lipids (Phospholipon 50, P50) and one of the selected PEs: Transcutol^®^ P (Trc), propylene glycol (PG), polyethylene glycol 400 (PEG), and Labrasol^®^ (Lab). The use of a mixture of PE/water (40% v/v) as hydrophilic phase, enabled the incorporation of 2 mg/mL of QUE in PEVs without any sign of drug precipitation during or after fabrication. Previous findings demonstrated that PEVs are powerful enhancers for dermal delivery, due to the synergistic effect of phospholipid vesicles and PE [4-8]. The latter may increase fluidity of the lipid portion of the stratum corneum, facilitating the delivery of the vesicle loaded-drug, and its diffusion through the skin. Quercetin-loaded PEVs were prepared, thoroughly characterized by size, surface charge, loading capacity, morphological and viscoelastic features. Moreover, their penetration capability and distribution through pig skin were assessed by Franz diffusion experiments and confocal microscopy, to give further evidence of the superior performances of PEVs.

## 2. Experimental Section

### 2.1. Materials

Soybean lecithin (Phospholipon^®^ 50, P50, with 45% phosphatidylcholine and 10–18% phosphatidylethanolamine) was kindly supplied by AVG (Milan, Italy) and Lipoid GmbH (Ludwigshafen, Germany). 1,2-dioleoyl-*sn*-glycero-3-phosphoethanolamine-N-(lissamine rhodamine B sulfonyl) ammonium salt (rhodamine-phosphoethanolamine, Rho-PE) was purchased from Lipoid GmbH (Ludwigshafen, Germany). Diethylene glycol monoethyl ether (Transcutol^®^ P, Trc) and caprylocaproyl macrogol 8-glyceride (Labrasol^®^, Lab) were a gift from Gattefossè (Saint Priest, France). Propylene glycol (PG), polyethylene glycol 400 (PEG), quercetin (QUE) and all the other products were of analytical grade and were purchased from Sigma-Aldrich (Milan, Italy).

### 2.2. Vesicle preparation

QUE (2 mg/mL) was dissolved in a PE/water solution (40% v/v) and added to the flask containing P50 (60 mg/mL). Lipids were left swelling in the solution overnight [9,10]. Sonicated vesicles were prepared by sonicating for 3 minutes (2 seconds on and 2 seconds off) the dispersions with a Soniprep 150 ultrasonic disintegrator (MSE Crowley, UK).

Each vesicle suspension was purified from the non-incorporated drug by exhaustive dialysis against distilled water at 4 °C for 1 h, using dialysis tubing (Spectra/Por^®^ membranes: 12–14 kDa MW cut-off, 3 nm pore size; Spectrum Laboratories Inc., USA). Incorporation efficiency (E%), expressed as the percentage of the amount of QUE initially used, was determined by high performance liquid chromatography (HPLC) after disruption of vesicles with 0.025% non-ionic Triton X-100. QUE content was quantified at 255 and 367 nm using a chromatograph Alliance 2690 (Waters, Italy). The column was a SunFire C18 (3.5 μm, 4.6 × 150 mm). The mobile phase was a mixture of acetonitrile, water and acetic acid (94.8:5:0.2, v/v), delivered at a flow rate of 1.0 mL/min.

### 2.3. Vesicle characterization

Vesicles were characterized by Transmission Electron Microscopy (TEM) for vesicle formation and morphology. A drop of the vesicular dispersion was applied to a carbon film-covered copper grid and stained with a 1% phosphotungstic acid. Then, samples were examined with a JEM-1010 (Jeol Europe, France) transmission electron microscope equipped with a digital camera MegaView III and Software “AnalySIS”, at an accelerating voltage of 80 kV.

The average diameter and polydispersity index (P.I.) of the samples (6 replicates) were determined by Photon Correlation Spectroscopy (PCS) using a Zetasizer nano-ZS (Malvern Instrument, UK). Samples were backscattered by a helium–neon laser (633 nm) at an angle of 173° and a constant temperature of 25 °C. The P.I. was used as a measure of the width of the size distribution: P.I. less than 0.4 indicates a homogenous and monodisperse population. Zeta potential was estimated using the Zetasizer nano-ZS by means of the M3-PALS (Phase Analysis Light Scattering) technique, which measures the particle electrophoretic mobility in a thermostated cell. All the samples were analyzed 24 h after their preparation.

### 2.4. Rheological studies

Steady shear tests and dynamic oscillatory tests were performed with a controlled strain and stress rheometer (Kinexus pro, Malvern Instruments, UK) equipped with a rSpace data acquisition software. Samples were allowed to rest for at least 300 seconds prior to analyses. Analyses were carried out using a double-gap concentric cylinder DG25 (also called Couette or Coaxial geometry). The test dispersion is maintained in the annulus between the cylinder surfaces. The double-gap configuration is useful for low-viscosity dispersions, as it increases the total area, and therefore the viscous drag, on the rotating inner cylinder, and increases the accuracy of the measurement.

Viscometry experiments were conducted in a shear range of 0.01–10 Pa. Frequency sweep tests were performed from 0.01 to 10 Hz, and at a shear stress of 0.5 Pa. The oscillatory parameters used to compare the viscoelastic properties of the different dispersions were the storage modulus (G&prime), which represents the elastic part of the response (where energy is stored and used for elastic recoil of the specimen when a stress is removed), the loss modulus (G″), which represents the viscous response (where energy is dissipated and the material flows) [11, and references therein]. All measurements were made in triplicate, at a constant temperature of 25 °C.

### 2.5. Ex vivo skin penetration and permeation studies

Experiments were performed non-occlusively using Franz diffusion vertical cells with an effective diffusion area of 0.785 cm^2^, and new born pig skin. One-day-old Goland–Pietrain hybrid pigs (∼1.2 kg) were provided by a local slaughterhouse. The skin, stored at −80 °C, was pre-equilibrated in physiological solution (0.9% w/v of NaCl) at 25 °C, two hours before the experiments. Skin specimens (n = 6 per formulation) were sandwiched securely between donor and receptor compartments of the Franz cells, with the stratum corneum (SC) side facing the donor compartment. The receptor compartment was filled with 5.5 mL of physiological solution, which was continuously stirred with a small magnetic bar and thermostated at 37 ± 1 °C throughout the experiments to reach the physiological skin temperature (*i.e.*, 32 ± 1 °C).

One hundred microliters of the tested vesicle suspensions, or coarse dispersions of P50, PE and free QUE, were placed onto the skin surface. At regular intervals, up to 8 h, the receiving solution was withdrawn and replaced with an equivalent volume of pre-thermostated (37 °C) physiological fresh solution, to ensure sink conditions. Withdrawn receiving solutions were analyzed by HPLC for drug content (as described in Section 2.2).

After 8 h, the skin surface of specimens was gently washed (3 times) with 1 mL of distilled water, then dried with filter paper. The SC was removed by stripping with adhesive tape Tesa^®^ AG (Hamburg, Germany). Each piece of the adhesive tape was firmly pressed on the skin surface and rapidly pulled off with one fluent stroke. Ten stripping procedures were performed consecutively. The method was previously validated by histological examination of stripped skin [6, and references therein]. Epidermis was separated from dermis with a surgical sterile scalpel. Tape strips, epidermis, and dermis were cut and placed each in a flask with methanol and then sonicated for 4 minutes in an ice bath to extract the drug. The tapes and tissue suspensions were centrifuged for 10 minutes at 10000 rpm, and then the supernatants were filtered and assayed for drug content by HPLC (see Section 2.2).

### 2.6. Confocal laser scanning microscopy

PEVs were made fluorescent by adding Rhodamine-phosphoethanolamine during the preparation. This labelling allows visualizing the penetration of the lipid bilayer materials through new born pig skin. (Trans)dermal study was carried out at the same conditions reported in paragraph 2.5. After 8 h of treatment, the skin specimens were washed, the diffusion area punched out, and rapidly frozen at −80 °C. Sections of skin (7 μm thickness) were cut with a cryostat (Leica CM1950, Barcelona, Spain) orthogonally (in the z axis) to the surface, and examined to investigate the fluorescent probe distribution in the different skin strata. Analyses were carried out using a FluoView FV1000 inverted confocal microscope (Olympus, Barcelona, Spain) equipped with a Ultraviolet/Visible light laser. Using a UPlanSApo 20x objective NA 0.75, images with a field size of 1024 × 1024 μm were generated. Rhodamine was excitated at 559 nm, and detected at 578 nm. The instrument offers a revolutionary synchronized laser scanning system, called the SIM Scanner: while one laser stimulates, the second laser simultaneously provides high-resolution imaging, which enables the acquisition of images showing the distribution of the marker among skin structures.

### 2.7. Statistical analysis of data

Data analysis was carried out with the software package R, version 2.10.1. Results are expressed as the mean ± standard deviation of at least six replicates coming from different preparation batches. Multiple comparisons of means (Tukey test) were used to substantiate statistical differences between groups, while Student's *t*-test was applied for comparison between two samples. Significance was tested at the 0.05 level of probability (p).

## 3. Results and Discussion

In this study, we prepared and characterized phospholipid vesicles containing high quantities of different PEs (40%) in the water phase, which is required to avoid quercetin (2 mg/mL) precipitation. Indeed, we aimed at investigating PEs capability to act as solubilizers for poorly water soluble QUE, and as penetration enhancers for improved (trans)dermal delivery of the drug, synergically with the vesicular carrier system. To this end, four different safe, biocompatible penetration enhancers, largely used in topical preparations, namely PG, PEG, Lab and Trc were used to formulate PEV dispersions, together with P50, a mixture of soy lipids.

### 3.1. Vesicle design and characterization

As mentioned, owing to the extremely poor aqueous solubility of quercetin, a novel delivery approach was designed; that is the preparation of vesicular dispersions. It is noteworthy that it was not possible to obtain conventional phospholipid liposomes (*i.e.,* P50 and QUE in water) with favorable characteristics, due to drug precipitation. On the contrary, using PE/water blends, novel PEVs were successfully produced.

**Figure 1.**
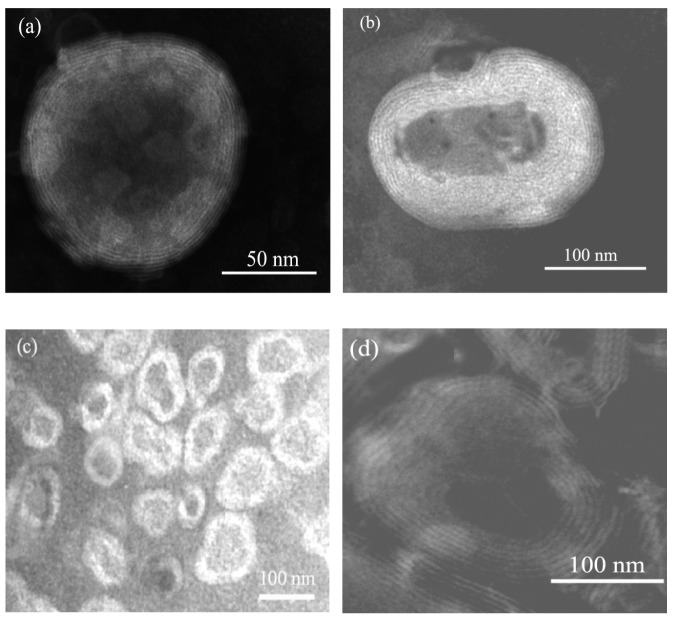
Negative stain electron micrographs of QUE-loaded PEVs prepared with: **(a)** propylene glycol, **(b)** PEG400, **(c)** labrasol, **(d)** transcutol.

Vesicle formation in the presence of the PE was confirmed by TEM (Figures 1a–d). PEVs were always multilamellar, showing an irregular and ovoidal shape, except PG-PEVs.

Mean size of PEVs, measured by PCS, was closely related to their composition (Table 1): vesicles containing Trc and PEG were approximately 2.5–3-fold larger than PG- and Lab-PEVs, being around 200 nm for the former, and 80 nm for the latter. This is clearly in accordance with TEM observations. The difference in size between empty and corresponding QUE-loaded vesicles was related to the composition of the samples: empty and QUE-loaded PEG-PEVs showed the same mean size; loaded PG- and Trc-PEVs were larger than the empty ones; loaded Lab-PEVs were smaller than the corresponding empty vesicles. PEVs were quite homogeneously dispersed and values were always repeatable. Zeta potential values were always highly negative (around −50 mV), indicative of a good storage stability against vesicle aggregation and fusion. Lab-PEVs showed a lower zeta potential (around −30 mV), with and without the drug. QUE incorporation into the vesicles at a percentage ranging from 48 to 75 was achieved by the prepared formulations (E%; Table 1), showing their good loading capacity, which was affected by the used PE. PEG-, PG- and Trc-PEVs showed the lowest E%, as a function of their high hydrophilicity (P_ow_ = 0.000015, 0.12 and 0.7, respectively).

The used PEs are characterized by a relatively high hydrophilicity and show good solvent capability for QUE. However, as a consequence of their molecular structure, the corresponding obtained PEVs exhibited somewhat different features (e.g. size, E%), presumably by virtue of specific interactions with the components of the formulations, related to peculiar properties, such as polarity, partition coefficient, and ability to interpenetrate the lipids.

### 3.2. Rheological behavior

**Figure 2.**
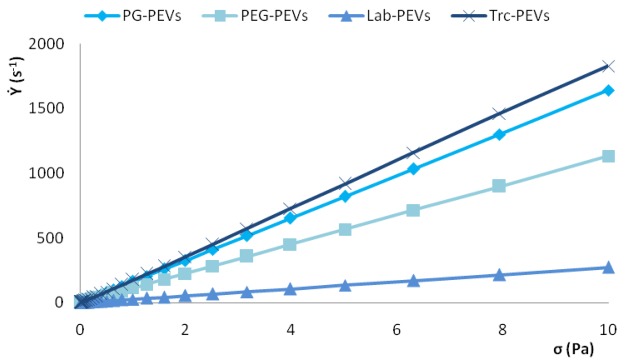
Linear scale plots of shear rate *vs* shear stress for QUE-loaded PEVs.

Macroscopic rheological properties of samples, such as viscosity, elastic or viscous moduli, depend on the strength of particle–particle interactions that occur at supramolecular level, and reflect changes in the microstructure of lamellar vesicles. The vesicle dispersions showed the rheological features normally observed in multilamellar vesicle dispersions. The behavior of the different PEVs on the shear rate against shear stress was first examined (Figure 2). For all samples, the shear rate increased when the shear stress increased, and shear viscosity (= shear stress/shear rate) was independent of the applied shear stress, as for Newtonian fluids. Indeed, in a Newtonian fluid, the relation between the shear stress and the shear rate is linear, the constant of proportionality being the coefficient of viscosity. The viscosity of PEVs was higher than that of water (1 mPa s): approximately 5, 6, 9 and 40 mPa s for Trc-, PG-, PEG- and Lab-PEVs, respectively. This important increase in viscosity is due to the existence of vesicular lamellar structure occupying a high hydrodynamic volume, especially for Lab-PEVs, which showed a smaller size, lesser number of bilayers, and an increased volume fraction occupied by the vesicles, closely related with the increase of viscosity.

**Figure 3.**
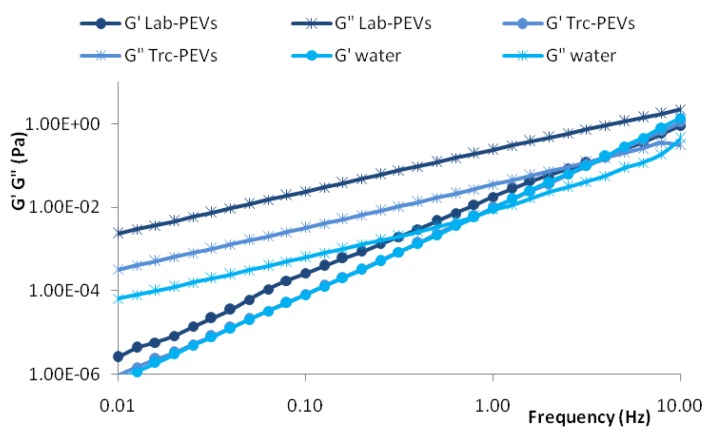
Frequency sweep spectra for PEVs: storage (G′) and loss (G″) moduli against frequency are shown.

In addition, we performed oscillatory frequency experiments to determine the storage (G′) and the loss (G″) response of the vesicular dispersions to the applied force. In Figure 3 representative mechanical spectra of samples are plotted against frequency, in comparison with water. It was found that Trc-PEVs, as well as PG- and PEG-PEVs, disclosed the same behavior for water, a purely viscous fluid. For these formulations, elastic modulus increased uniquely due to the inertia effect, while the viscous modulus was a little higher than that of water, as evidenced by the viscometry study. In contrast, Lab-PEVs showed a higher loss modulus (by about 1 order of magnitude with respect to water) and a storage modulus only slightly higher than that of water, indicating the presence of an elastic component, even if the viscous one predominated.

Further, it was evident that the loss modulus (G″) was significantly higher (by about 3 orders of magnitude) than the storage modulus (G′) throughout the employed frequency range, confirming the viscous nature of PEVs. The smaller magnitude of the elastic modulus indicates weak particle–particle interactions. Therefore, the samples showed the typical behavior of diluted spherical multilamellar vesicle dispersions, where the storage modulus is lower than the loss modulus (G′ < G″) [12], indicating the viscous nature of the samples. Each sample showed different values of viscosity, storage and loss moduli because the different PEs in the vesicle dispersions caused different degrees of swollen lamellar phase.

These results are consistent with the nature of the tested samples: they are diluted dispersions, which behave as ideal Newtonian fluids that simply flow when subjected to a stress, as they are non-structered systems with weak vesicle interactions. On the other hand, the presence of labrasol probably makes the vesicular bilayer more deformable, resulting in an increase of the elastic component.

### 3.3. Ex vivo skin penetration and permeation studies

**Figure 4.**
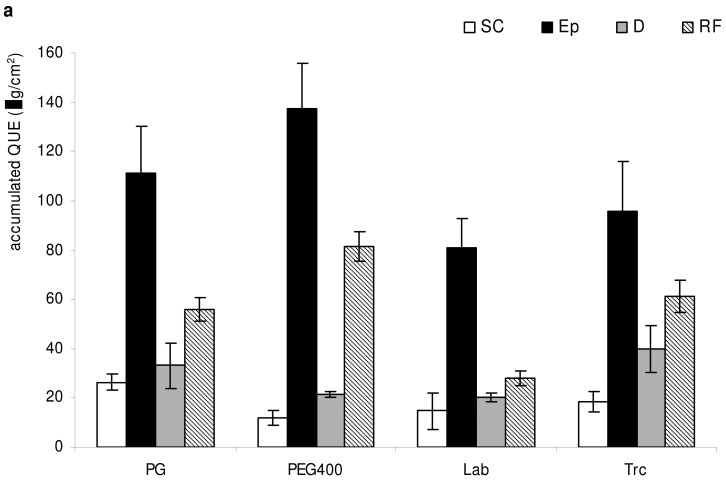

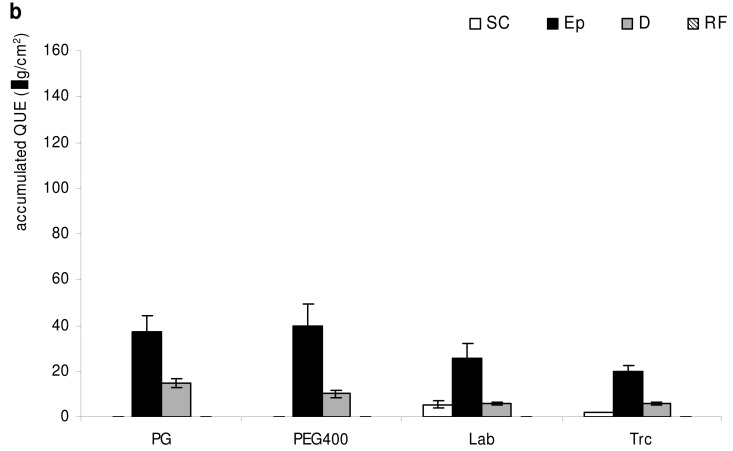
Determination of QUE deposition into pig skin layers (SC, stratum corneum; Ep, epidermis; D, dermis and RF, receptor fluid) after 8-h non-occlusive treatment: **(a)** QUE loaded in PEVs, **(b)** coarse dispersions of P50, PE and non-incorporated QUE. Each value is the mean ± S.D. of six experimental determinations.

The skin penetration ability of quercetin loaded in PEVs was probed by *ex vivo* Franz diffusion studies on new born pig skin. The amount of drug accumulated into stratum corneum, epidermis, and dermis is expressed as the percentage of the drug applied onto the skin. As illustrated in Figure 4a, all PEVs promoted QUE deposition in the three main skin strata, showing the same behavior in all samples: the lowest drug accumulation in the stratum corneum, the highest in the epidermis, slightly higher in the dermis than in stratum corneum, and significantly lower in receptor fluid than in epidermis. In particular, PG- and PEG-PEVs allowed the highest drug accumulation into (∼170 μg/cm^2^) and through (∼5 μg/cm^2^) the skin. Vesicle ability to deliver QUE to the skin seems to decrease when viscosity and loss modulus of dispersion increase (Lab-PEVs), probably because the volume fraction occupied by the vesicles enhances, causing an improvement of vesicle–vesicle interactions.

To clarify the role of the studied vesicles in (trans)dermal drug delivery, and in particular to elucidate if they act as carriers or penetration enhancers, coarse dispersions of the same composition used to produce vesicles were tested, too. As can be seen in Figure 4b, when the coarse dispersions were used, the drug accumulated in the skin layers was lower: about 6-fold for PG-, PEG- and Lab-PEVs, 20-fold for Trc-PEVs, than that obtained with the corresponding QUE-loaded vesicular formulations, or even nil in the stratum corneum in the case of PG- and PEG-PEVs treated skin. None of the coarse dispersions with free QUE was found to determine a systemic drug permeation. Results obtained confirm PEVs' capability to behave as true carriers, and not simply as penetration enhancers [5,6].

### 3.4. Confocal microscopy examination

**Figure 5.**
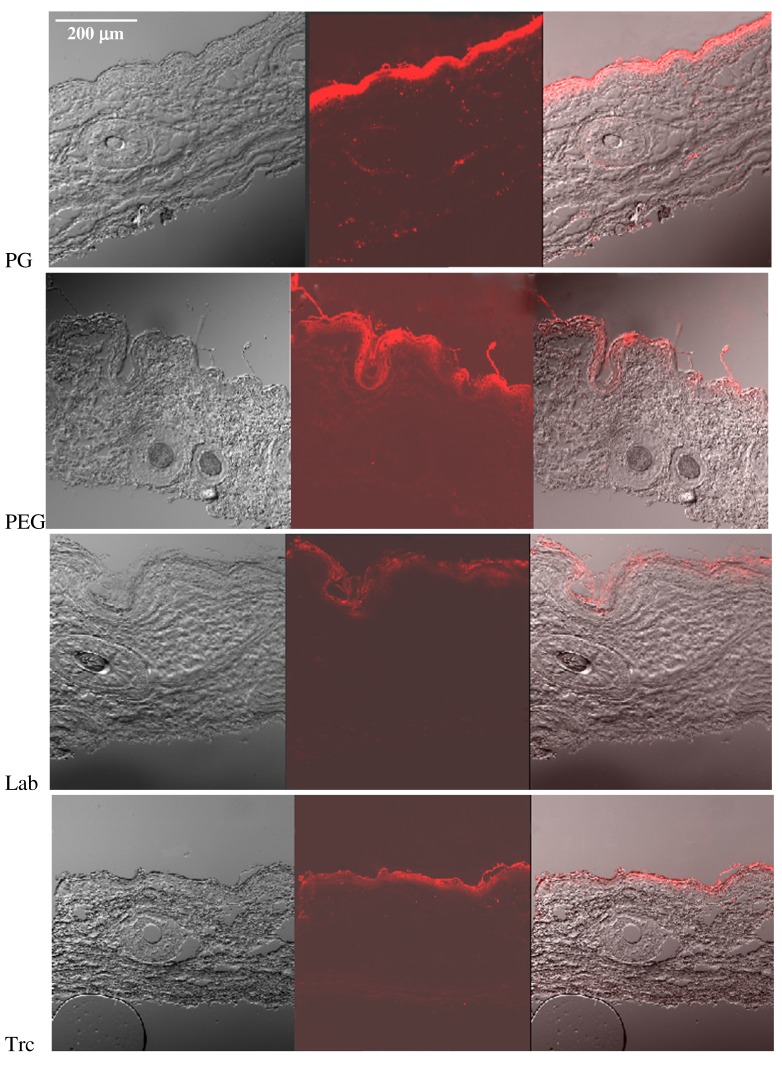
CLSM of cross-sections of new born pig skin treated for 8 h with PEVs labeled with rhodamine-phosphoethanolamine. PG, PEG, Lab and Trc were used to formulate PEV dispersions.

PEVs were labeled with Rho-PE and applied onto pig skin for 8 h. The fate of the fluorescent bilayer materials through new born pig skin was visualized using the CLSM technique. Mechanical cross-sections were made othogonally to the skin surface, to obtain a good estimation of distribution pattern of fluorescent phospholipids. These cross-sections provided on a single focal plane a simultaneous visualization of stratum corneum, viable epidermis and dermis. Figure 5 consists of four cross-sections (∼400 μm) of pig skin treated with labeled PEVs containing alternatively PG, PEG, Lab or Trc. Images indicate a high accumulation of Rho-PE in the SC (∼70 μm in depth), since most of the vesicles penetrate into the stratum corneum and fuse with the intercellular lipids. Indeed, phospholipid vesicles disorder the complex structure of the upper intercellular lipid sheets, but also cause an occlusive effect, which helps hydratation of the keratin layer, resulting in an increased penetration. The free drug could easily penetrate into the viable epidermis, but also drug-loaded intact vesicles are facilitated in their passage through the skin, carrying and delivering the drug to the viable epidermis and dermis. Overall, fluorescence in the four skin cross-sections was always higher in SC than in epidermis and dermis; PEG-PEVs treated skin showed a high fluorescence also in epidermis; PG- and Trc-PEVs promoted Rho-PE accumulation in the dermis, whereas Lab-PEVs treated skin specimen showed only a slight fluorescence in the dermis. In light of these findings, PEVs seem to be able to penetrate the skin intact, reaching the lowest SC layers where they form a depot from which the drug can be released [5,13,14].

## 4. Conclusions

In this study we used a high amount (40%) of hydrophilic PEs to facilitate QUE incorporation into phospholipid bilayer, and to avoid QUE precipitation in vesicle dispersions. Results underline the ability of PEs (propylene glycol, polyethylene glycol 400, labrasol and transcutol) to improve drug solubility in vesicle dispersion, but above all, to have a synergic effect with phospholipids as penetration enhancers, which make PEVs potent nanocarriers for QUE skin delivery.

## Figures and Tables

**Table 1.** Characteristics of empty and QUE-loaded PEVs: mean diameter (MD), polydispersity index (P.I.), zeta potential (ZP) and incorporation efficiency (E%). Each value represents the mean ± S.D., n = 6.

		**MD (nm)**	**P.I.**	**ZP (mV)**	**E (%)**
P50/PG	Empty	52±10	0.36	−51±7	
	QUE	83±10	0.35	−63±4	57±8
P50/PEG	Empty	193±5	0.30	−51±2	
	QUE	190±4	0.31	−58±2	48±7
P50/Lab	Empty	135±10	0.27	−21±4	
	QUE	86±5	0.29	−32±3	75±9
P50/Trc	Empty	156±6	0.19	−51±6	
	QUE	226±5	0.28	−49±5	59±8
